# Quarantine: A Period of Self-discovery and Motivation as Medical Student

**DOI:** 10.31729/jnma.5005

**Published:** 2020-07-31

**Authors:** Suzit Bhusal, Aliska Niroula, Rijan Kafle

**Affiliations:** 1 Kathmandu Medical College and Teaching Hospital, Sinamangal, Kathmandu, Nepal; 2 B.P. Koirala Institute of Health Sciences, Dharan, Nepal

## Abstract

The ongoing nationwide lockdown due to the global pandemic COVID-19 started from March 24, 2020, in Nepal. Lots of students are in dilemma about how to utilize this time to make it more productive. To live a good life, we must be able to balance our life in general so, we can use this time to discover ourselves. It is equally important to adjust to the global pandemic and help locally to combat the current situation. This difficult time demands resilience. This article focuses on some ideas to discover ourselves and develop resilience within us.

## INTRODUCTION

Coronavirus: the worldwide attention-seeker of 2020. The coronavirus disease 2019 (COVID-19) is an acute respiratory disease that is caused by a novel coronavirus (SARS-CoV-2, previously known as 2019-nCoV). It has spread throughout China. On 30 January 2020, the World Health Organization (WHO) officially declared the COVID-19 epidemic as a public health emergency of international concern.^1^

The word quarantine is used to describe isolation to prevent the spread of infection comes from the Latin word “Quaranta,” meaning 40, because the isolation lasted for 40 days in the old port of Dubrovnik; the world's first quarantine. Since the fourteenth century, quarantine has been the cornerstone of a coordinated disease-control strategy, including isolation, sanitary cordons, bills of health issued to ships, fumigation, disinfection, and regulation of groups of persons who were believed to be responsible for spreading the plague infection.^2^

As a medical student, most of us have very unhealthy or unbalanced schedules. So, we can use this time to discover ourselves. It is important to be ourselves, but it is more important to know ourselves. As we can discover ourselves after taking a break to think and assess our life. Finding peace in silence can help us a lot to discover about ourselves. And, this is a boon of the ongoing lockdown.

## SELF-DISCOVERY

### 1. A habit of forming a routine behavior

Forming a routine can take a long time and is highly variable between individuals.^3^ As in quarantine, we can use our days to develop ourselves personally to form a regular habit which we were planning to form but got distracted due to any reasons.

### 2. A habit of self-reflecting

Forming a habit of writing a daily journal is a way of reflecting ourselves. It has been recognized as a method designed to enhance reflection, facilitate critical thought, express feelings in writing about problems encountered during clinical experiences, and practice writing summaries, objectives, and focused arguments.^4^ Living in social distancing is stressful so we can vent our sad moments out, add positivity in our life and be motivated by maintaining a daily journal book.

### 3. Meditation and Yoga

Yoga is characterized by balance, health, harmony, and bliss. By practicing yoga, a person is supposed to reach a state of mental equanimity, where responses to favorable or unfavorable external events are well under the individual's control, and responses are moderate in intensity.^5^ The outbreak of COVID-19 may be stressful for most of us, so, we can cope with this situation by practicing yoga and meditation.

### 4. Stretching exercises

Aerobic exercises, including jogging, swimming, cycling, walking, gardening, and dancing, have been proved to reduce anxiety and depression.^3^ These improvements in mood are proposed to be caused by exercise-induced increase in blood circulation to the brain and by an influence on the hypothalamic-pituitary-adrenal (HPA) axis and, thus, on the physiologic reactivity to stress.^6^ Acute bouts of physical activity are associated with short-term increases in a positive mood, even on days when negative daily events occur. Some activities may be more effective than others in boosting positive affect, however. In particular, aerobic activities appear to elevate a positive mood more than do nonaerobic activities, including yoga and body conditioning. These all lead to a tough mentality which is what time demands. One must keep in mind that all these activities must be done indoors.^7^

### 5. Sharing with needy ones

We can share our knowledge, food, water, emotions, experiences, etc. to people around us. Food offering may, therefore, be support behavior in and of itself, but can also serve as a facilitator of other forms of support.^8^ We can face scarcity of water during summer and hence water sharing can be a good way to help people living in those areas. Water sharing offers insight into every day and, at times, invisible ties that bind people and households with water and to one another.^9^

### 6. Take a "nature" break

Exposure to natural environments, like parks, forests, and fields, is associated with better health and an increase in self-resilience. Spending as little as an hour in contact with nature (versus in an urban setting) appears to have a restorative effect, both increasing positive emotion and decreasing negative emotion and physiological arousal. Moreover, taking a break in an outdoor setting like a garden may improve concentration, even compared to spending a similar amount of time in a pleasant indoor setting.^7^

Also, it is equally important to adjust to the global pandemic and help locally to combat the current situation. Hence, self-motivation is the capacity to recover quickly from tough situations. It is by far one of the most important characteristics a person must have. This difficult time demands resilience. To overcome this we must be tough. However, self-motivation is innate to a person but it can be increased by doing different activities.

## SELF-MOTIVATION

### 1. Feeling happy and being optimistic

Relevant to the current discussion, individuals who are happy and optimistic are tough. They can take difficult situations more easily than other people and this what this time demands.

Even a relatively small increase in participation in pleasurable activities can provide a boost in wellbeing. Individuals who are instructed to engage in more frequent pleasurable activities of their choosing, show more resilience behaviors.^7^

### 2. Listening podcasts

Podcasts are audio programs that we listen to through our phones and are one of the best ways to become smarter in your spare time. They have become increasingly popular, particularly among young people. Although many podcasts are for entertainment rather than educational purposes, The BMJ has highlighted their role in medical education.^10^ For those who prefer learning by listening, podcasts are a great way to complement traditional learning methods. They also enable learning on the go or while doing other things. Some podcasts adopt a case based approach with clinical examples, whereas others focus on one disease or topic. While listening to podcasts one can be involved in other activities like running, cooking, etc. As per studies podcast of 30 minutes seems to be very useful for students. Some of the podcast suggestions are:

- The Undifferentiated Medical Student
- Old PreMeds Podcast
- Academic Medicine Podcast
- Instant Anatomy
- JAMA Clinical
- Board Rounds

### 3. YouTube

YouTube can be a great alternative or supplement to books and lectures. One can find an enormous amount of content relevant to medical studies. Different sites he/she can look up to in YouTube are:

- Armando Hasudungan^11^
- Khan Academy^12^
- Najeeb lectures^13^
- Osmosis^14^

### 4. Physical exercise and home workout

Since during quarantine, we are mostly inside our houses we can try some of the home workouts using some apps like Running-runtastic, couch to 5k, 10k, strava. We can listen to lots of podcasts to keep us motivated while exercising.

### 5. Personal development

Developing ourselves personally makes us more confident, happy, and productive. It helps us to understand ourselves and create our own identity which helps us in our career. The self-development coaching program showed a short-term improvement in depression and anxiety.^15^ Some of the skills we can develop are communication skills, problem-solving skills, adaptability, etc. These skills help us personally as well as professionally.

### 6. Watching movies, series shows, etc.

Watching movies and series are our very good time passing activities; we can make these activities productive by watching movies and series that aware as well as motivates us. We can watch some documentaries, biopics, a movie in which a person is struggling with some disease: this helps us to remember the clinical features of the disease and maybe some of the useful treatment, etc.

### 7. Going through medical journal

Journal is a magazine or newspaper that deals with a particular subject or professional activity. As a medical student being acquainted with the topics from time to time is very important. Journals provide a platform for students where they can learn new things. Journals like The BMJ, Geneva foundation for medical and research; New England Journal of Medicine, British Medical Journal, etc. are always helpful for students to go through various topics.

### 8. Updating ourselves on the ongoing pandemic

As COVID-19 is an emerging disease, there are lots of researches being done on it. We should update ourselves on this ongoing pandemic by reading new articles on the recent research of the virus and disease pattern, newsletter by the World Health Organization, e-newspapers, etc. There are lots of misleading news on the internet so we should check the source of the information. It helps us to be concerned about the upcoming crisis and work accordingly.

### 9. Alternative platforms

While video and audio learning platforms are relatively familiar to many medical students, other innovative platforms exist. Some revolve around creating visual representations to help memorize information. Sources to look up to as an alternative platform are:

- Picmonic (picture + mnemonics): It helps students to retain and recall the large number of facts which are studied in medical school.^13^
- Anki flashcards: It helps in spaced repetition which increases the rate of memorization.
- 3d anat teach me anatomy: It helps in creating a good concept in anatomy which most of us forget if it is not revised timely.^13^
- Online courses: We can follow Coursera, Udemy, WebMD, etc.

## Conflict of Interest

**None.**
